# High Resolution 3‐T Magnetic Resonance Tractography Cannot Localise Conduction Block in Multifocal Motor Neuropathy With Conduction Block

**DOI:** 10.1111/jns.70164

**Published:** 2026-09-13

**Authors:** Ryan Y. S. Keh, Laura Zambreanu, Stephen Wastling, Carolynne M. Doherty, Hamza A. Salhab, Chandrashekar Hoskote, Sachit Shah, Julian Blake, Aisling S. Carr, John Thornton, Jasper M. Morrow, Michael P. Lunn

**Affiliations:** ^1^ Centre for Neuromuscular Diseases, National Hospital of Neurology and Neurosurgery, Queen Square University College London Hospitals NHS Foundation Trust London UK; ^2^ Manchester Centre for Clinical Neurosciences, Salford Royal Hospital Northern Care Alliance NHS Foundation Trust Manchester UK; ^3^ Lysholm Department of Neuroradiology, National Hospital of Neurology and Neurosurgery, Queen Square University College London Hospitals NHS Foundation Trust London UK; ^4^ Department of Brain Repair and Rehabilitation, Institute of Neurology University College London London UK; ^5^ Department of Clinical Neurophysiology Norfolk and Norwich University Hospital Norwich UK; ^6^ Department of Neuromuscular Disease, Institute of Neurology University College London London UK

**Keywords:** diffusion tensor imaging, MRI, multifocal motor neuropathy

## Abstract

**Background and Aims:**

Definite diagnosis of multifocal motor neuropathy with conduction block (MMNCB) requires identification of electrophysiological motor conduction block (CB), which is sometimes challenging to demonstrate. Magnetic resonance (MR) neurography is recommended as a supportive diagnostic criterion. Brachial plexus short *tau* inversion recovery (STIR) hyperintensity is described in MMNCB but the utility of tractography (DTI) is less well understood. We sought evidence for the utility of DTI and STIR parameters of MMNCB forearm nerve segments with clear NCS‐demonstrated CB, which could be applied to less accessible nerves.

**Methods:**

Ten MMNCB individuals with definite CB and 10 healthy controls underwent dedicated forearm 3‐T MRI with DTI and STIR. Axial, radial and mean diffusivity (AD, RD and MD) and fractional anisotropy (FA) were calculated. DTI profiles were visually inspected for possible abnormalities, and pooled values of individual diffusion parameters were analysed for group differences. STIR sequences were reviewed for areas of hyperintensity. MRI and defined NCS abnormalities were correlated.

**Results:**

Composite nerve segment profile inspection showed occasional patterns (elevated AD/MD/RD, reduced FA) in regions of CB, but indistinguishable changes were also seen in MMNCB nerves without CB and controls (sensitivity 28.6%–42.9%, positive predictive value 44.4%–54.5%). Pooled diffusion parameter data identified no statistically significant group differences. Some MMNCB nerves demonstrated focal STIR hyperintensity poorly correlating with CB.

**Interpretation:**

Existing DTI and STIR techniques cannot identify useful characteristics of MMNCB pathology in forearm nerves. Whilst statistically significant group differences can be identified, inspection of individual images cannot identify consistently found abnormalities, and the differences are too small to be clinically useful.

## 1 Introduction

There has been sustained and growing interest in magnetic resonance imaging (MRI) techniques as a diagnostic tool for neuromuscular diseases. Improvements in resolution offer the possibility that MRI could directly correlate structural changes with pathology similar to changes that might be seen in the central nervous system. Because of this, MRI has been included in several diagnostic criteria sets for neuropathies [1, 2].

However, even the most up‐to‐date MRI neurography has limitations as a diagnostic tool. Pathological MRI changes in neuromuscular conditions are often non‐specific and cannot easily differentiate between various diffuse neuropathies [3].

Multifocal motor neuropathy with conduction block (MMNCB), also known as multifocal motor neuropathy (MMN), is an inflammatory neuropathy with a typically good response to immunomodulatory treatment, including immunoglobulin [2]. A definite diagnosis of MMNCB requires a combination of clinical characteristics and the hallmark electrophysiological abnormality of motor conduction block (CB) [2]. CB can be hard to electrophysiologically identify despite extensive nerve conduction studies (NCS), as evaluable nerves are dependent on the accessibility of the nerve to surface stimulation. Therefore, identifying very proximal CB using electrophysiological methods is challenging.

Because of these limitations, supportive tests might be used to aid the diagnosis of MMNCB where clear demonstration of CB on NCS is not possible as suggested in the European Federation of Neurological Societies/Peripheral Nerve Society (EFNS/PNS) MMN Guidelines [2]. These include identification of ganglioside autoantibodies against GM1, elevated cerebrospinal fluid (CSF) protein levels, objective response to appropriate immunomodulatory treatment and MRI of nerve, in particular, the brachial plexus.

MRI in patients with MMNCB exemplifies both the utility and limitations of MRI techniques in diagnosing neuropathies [4]. Brachial plexus MRI abnormalities have been described in MMNCB in T2 [4, 5, 6, 7] and T2‐short *tau* inversion recovery (STIR) sequences [8], but the sensitivity and specificity have not been quantified. The abnormal MRI characteristics in MMNCB (hyperintensity, hypertrophy and asymmetry of signal change) also occur in other inflammatory neuropathies including chronic inflammatory demyelinating polyradiculoneuropathy (CIDP) and POEMS syndrome. Interrater reliability in quantitatively interpreting existing brachial plexus MRI is also low [9]. These limitations indicate a need for improvements in imaging tools for MMNCB diagnosis.

Diffusion tensor imaging (DTI) tractography is an established MRI approach for brain imaging with expanding research and clinical applications [10]. Fibre tracts are formed by nerve axons and associated cells which directionally restrict water diffusion. DTI quantifies water diffusion at each measured point, allowing derivation of a range of parameters [10], including fractional anisotropy (FA), axial diffusivity (AD), mean diffusivity (MD) and radial diffusivity (RD), representing normal integrity or disruption of anatomical tracts by axonal degeneration, inflammation, demyelination or other processes.

The utility of DTI in peripheral nerve disorders is not as well‐understood as in the brain. Early studies suggest that DTI of the brachial plexus might distinguish between CIDP, MMNCB and segmental spinal muscular atrophy (sSMA) but is inferior to ultrasound [11]. Previous studies indicated that DTI characteristics of the upper limb nerves may differ between MMNCB, amyotrophic lateral sclerosis (ALS) and healthy controls (HCs) [12, 13]. In these studies, median, ulnar and radial nerves were analysed together as a group with insufficient information to determine if there were DTI differences between nerves with and without CB.

The objective of our study was to determine whether we could identify differences in MRI DTI parameters of forearm nerve segments combined with clearly demonstrated CB on NCS in *individuals* with MMNCB, to identify intrinsic differences of MMNCB nerves compared to controls. This aim, if successful, was with a long‐term view to explore the potential of DTI as an indicator of CB in less accessible regions of the peripheral nervous system.

## 2 Materials and Methods

We included individuals with MMNCB from the National Hospital for Neurology and Neurosurgery (NHNN) MMNCB patient cohort with defined CB in forearm nerves. Adult individuals (over 18 years of age) with ‘definite MMN’ by EFNS/PNS criteria [2] were studied. Individuals with coexisting other known peripheral nervous system disorders of the upper limb (including disorders of cervical nerve roots, brachial plexus or upper limb peripheral nerves of any cause) were excluded. Twelve individuals were screened for inclusion. Contemporaneous NCS were performed on the median, ulnar and radial nerves in both upper limbs in all MMNCB patients with ‘inching’ to identify and clearly localise CB, which was characterised as ‘definite motor CB’ and ‘probable motor CB’ in accordance with EFNS/PNS criteria [2]. Two individuals did not meet criteria for CB in at least one forearm nerve at the time of screening and were removed.

To improve our confidence that CB was present at time of imaging, subjects underwent the MR neurography protocol as soon as feasible after NCS. In eight cases, this was on the same day as NCS, and the remaining two were imaged 4 and 28 days after NCS, respectively. Recruited MMNCB patients had been clinically stable with no recently resolved forearm CB that might be expected to confound imaging results. Ten age‐ and sex‐matched HCs were imaged identically.

**FIGURE 1:**
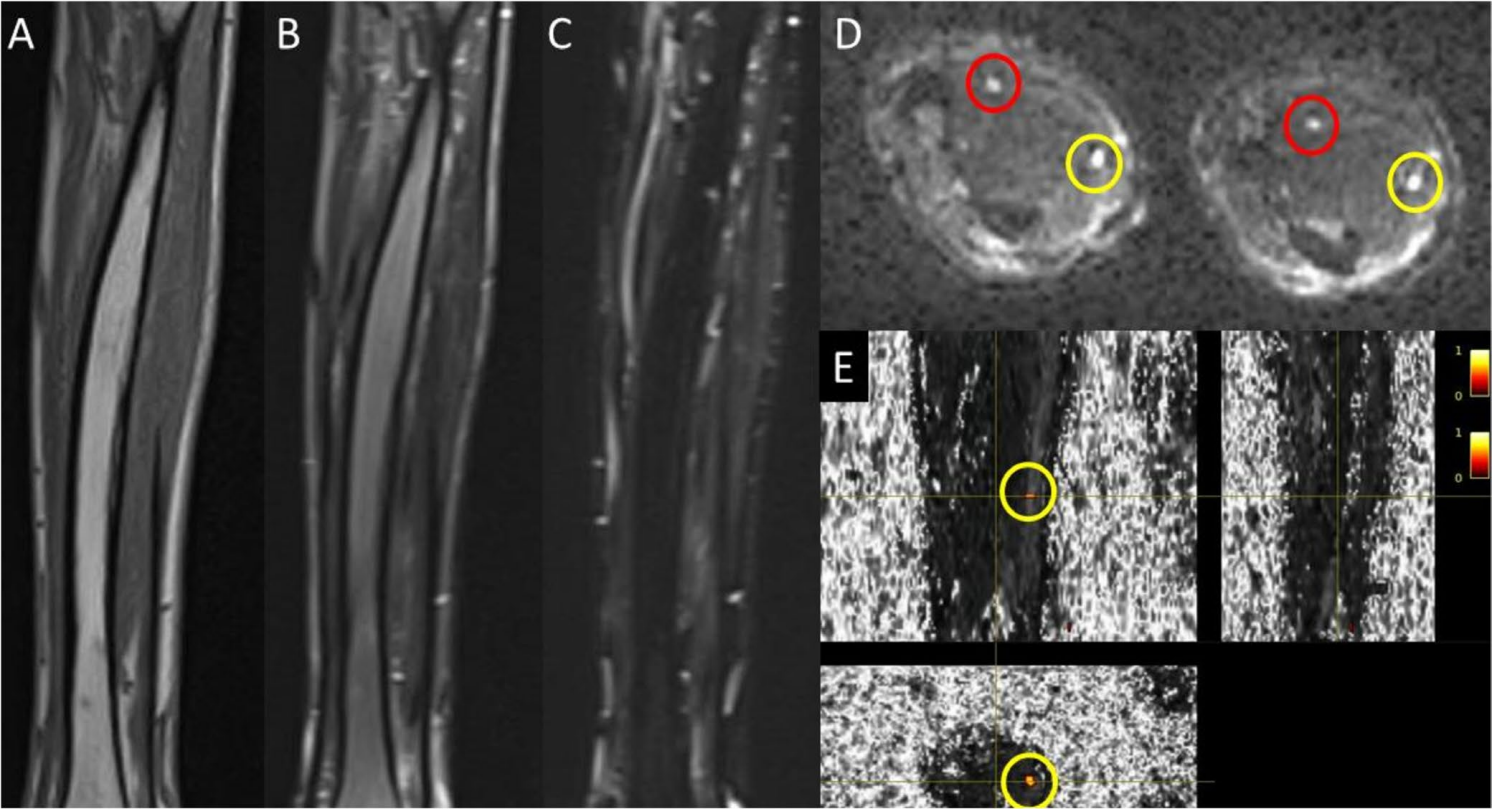
MR Neurography image acquisition. Coronal MR imaging of the right forearm (A: T1‐weighted Vibe Dixon, B: T2‐weighted, C: T2‐STIR‐weighted). (D) Axial DTI of the distal forearm allowing for the identification of median (red circles) and ulnar (yellow circles) nerves. (E) Region of interest drawn on right ulnar nerve (yellow circles) to be used to seed tractography.

All participants underwent a dedicated forearm magnetic resonance (MR) neurography protocol. Images of both forearms were acquired using a 3 T MAGNETOM Skyra (Siemens Healthineers) MR system using an 18‐channel phased array coil wrapped around the forearm. The subjects were positioned on their side with the forearm of interest positioned by their side as close to the centre of the scanner table as possible. Patients were re‐positioned to lie on their opposite side when scanning their other arm. Diffusion weighted imaging was performed using a single‐shot spin‐echo echo‐planar imaging sequence with the following parameters: 65 axial slices with 4 mm slice thickness, 240 by 120 mm field‐of‐view, 160 × 160 acquisition matrix resulting in 1.5 mm in‐plane voxel size, GRAPPA 2, 1 average, TR 7.5 s, TE 80 ms, fat‐saturation (strong), bandwidth 1250 Hz/pixel, *b*‐values 0 and 800 s/mm^2^, 20 diffusion directions. Two series were acquired, the first with phase‐encode direction anterior–posterior and the second with phase‐encode direction posterior–anterior. Figure 1 demonstrates an example of the main steps in image acquisition.

Diffusion data were analysed using MRtrix 3.0.2 [14]. Images were denoised [15, 16, 17], Gibbs ringing artefacts removed [18], corrected for susceptibility and eddy current induced distortions and subject movements [19, 20]. The response function for spherical deconvolution was estimated [21, 22], the images up sampled to 1.3 mm^3^ isotropic voxel size. The diffusion tensor was estimated by iterative weight least squares [23] and the first‐eigenvector, AD, MD, RD and FA calculated [24, 25]. Fibre orientation distributions were estimated by spherical deconvolution [26]. Regions of interest were drawn on the FA maps in the proximal, middle and distal third of the forearm nerves (median, ulnar and radial) by a single non‐blinded rater, and used to seed the tractography. The MRtrix *tckgen* command [14] was used to produce streamlines based on this seeding until a maximum of 5000 streamlines were produced, or until failure after 5000,000 attempts. Each nerve was divided into thirds based on direct measurement of distance between the elbow and wrist on the FA map, dividing this into three segments similar to the approach used in prior studies [12], and the same approach was used to localise CB on NCS.

Tractography was performed using two methods, firstly deterministic tensor tractography (tensor) [27] and secondly second‐order integration over fibre orientation distributions (iFOD2) [28]. Profiles of the diffusion parameters along the length of the nerve were extracted using an in‐house script (in Python 3.7) [29]; specifically, the mean and standard deviation of each of the diffusion parameters (weighted by the number of streamlines per voxel) were calculated for each slice.

The DTI profiles for each nerve were combined and visually inspected for any consistently found patterns of abnormality within known areas of CB on NCS.

We also analysed the pooled values of individual nerve segment FA, AD, MD and RD to determine if there were statistical differences between nerves with and without CB, as well as MMNCB and HC nerves. The distal median and proximal radial segments were chosen for this purpose as a representative subset of the larger dataset, with more than 1 million observations available for each parameter. STIR images were obtained with the following parameters: 144 sagittal slices with 1 mm slice thickness, 250 × 144 mm field‐of‐view, GRAPPA 2, 2 average, TR 3 s, TE 248 ms, fat‐suppression (none) and bandwidth 465 Hz/pixel. STIR sequences of the forearms were reviewed by two blinded specialist neuroradiologists with expertise in MR neurography for any structural correlates for CB. Neuroradiologists were blinded to subjects and groups, and images were reviewed concomitantly, with consensus decision on normality/abnormality where equivocal changes were noted. At the planning phase, the intention was to perform semi‐quantitative evaluation of the degree of nerve STIR hyperintensity and thickening, sub‐dividing changes into normal, mild/moderate and severe categories. However, this was not feasible in practice because of the notable degree of inter‐subject variability in image quality and the overall conspicuity of the nerves despite optimal imaging. Instead, the presence or absence of nerve STIR hyperintensity was evaluated qualitatively using the approach below.

The median and ulnar nerves were individually visually inspected (radial nerves not assessed as visualisation of radial nerves was inconsistent across subjects). All forearm nerves were first inspected for normality or identifiable abnormalities. Following this, each individual nerve (right and left median and ulnar) was individually visualised throughout its course. Where abnormalities were thought to be present, they were reported as focal, multifocal or diffuse. Focal abnormalities were defined as a single segment of abnormal signal measuring less than 5 cm. Diffuse abnormalities were defined as a contiguous segment of abnormal signal over 50% of the length of the nerve. Multifocal abnormalities were defined as two or more non‐contiguous signal abnormalities along the course of the nerve. For focal or multifocal abnormalities, the location along the course of the nerve was reported as involving proximal, middle or distal thirds of the nerve.

### 2.1 Statistical Analysis

Data analysis was performed using R v4.0.3 (R Core Team) [30].

Kruskal–Wallis test was used for testing of data acquisition quality comparing nerve segments to completeness of data collection, with Dunn tests for nonparametric multiple pairwise comparisons with Bonferroni correction where Kruskal–Wallis *p* < 0.05. For the large‐volume datasets involving pooled diffusion parameter values for individual nerve segments, mean FA, AD, MD and RD values for each individual nerve segment were compared using Kruskal–Wallis and Mann–Whitney *U* tests.

### 2.2 Ethics

Ethical approval was obtained from the London—Queen Square Research Ethics Committee: 10/H0716/83 and written informed consent was obtained from all study participants.

## 3 Results

Table 1 summarises the clinical and NCS characteristics of the subjects. Equal proportions of male and female participants were recruited for MMNCB and HC groups, and there were no significant age differences between groups (*p* = 0.73). Eight of the 10 MMNCB patients were on maintenance IVIg whereas two had no maintenance treatment. Further details of the areas of CB identified on each MMNCB patient are as detailed in Table S1.

**TABLE 1:** Clinical characteristics of recruited subjects.

	MMNCB	HC
Total number of subjects	10	10
Number of female subjects (%)	5 (50%)	5 (50%)
Median age (SD)	47 (15.7)	48.5 (11.9)
Number of subjects actively receiving Ig treatment (%)	8 (80%)	
Number of nerves with CB	One nerve—7 Two nerves—1 Three nerves—2	
Site of CB	Ulnar only—4 Median only—2 Radial only—1 More than one nerve—3	

Abbreviations: CB, conduction block; HC, healthy controls; Ig, immunoglobulin; MMNCB, patients with multifocal motor neuropathy with conduction block; SD, standard deviation.

### 3.1 Data Quality Analysis

**FIGURE 2:**
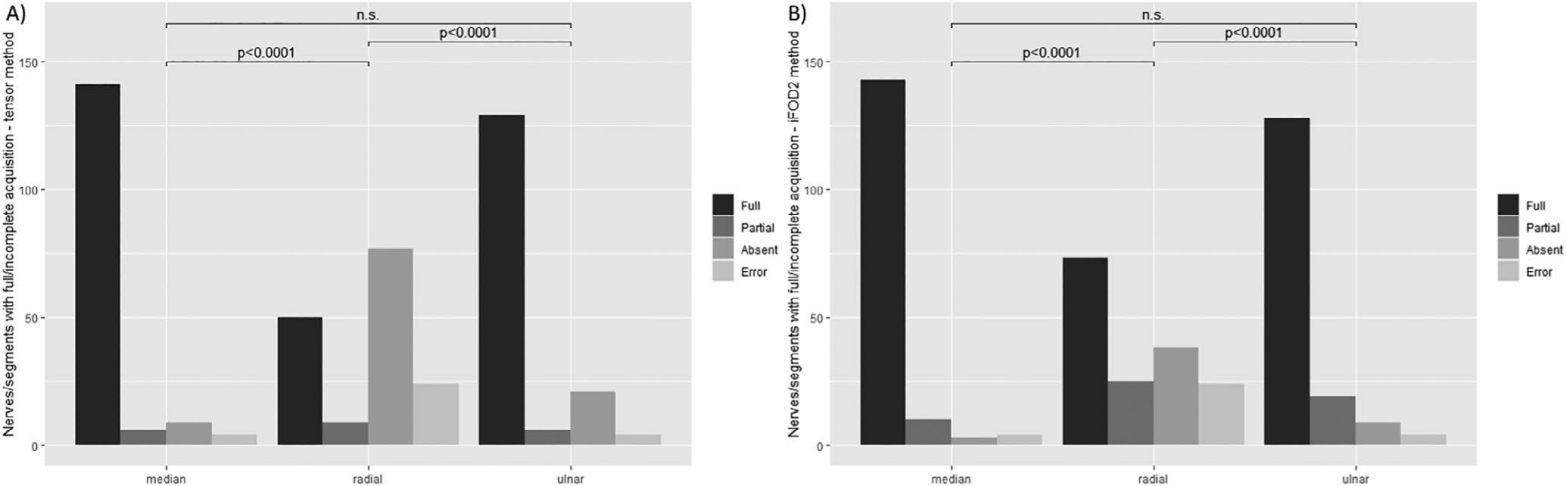
Proportions of nerve segments within the entire cohort from the median, radial and ulnar nerves with full, partial or no (absent/error) acquisition using the Tensor (A) and iFOD2 (B) algorithms. Full acquisition was achieved where the algorithm successfully seeded 5000 streamlines. Partial acquisition was defined as the algorithm seeding anywhere between 1 and 4999 streamlines after reaching the maximum number of sampling attempts. Absent acquisition and errors resulted in no diffusion parameter information for the given nerve segment. n.s., non‐significant (*p* ≥ 0.05).

As a quality control measure, we analysed the completeness of tractography from individual nerve segments to determine whether data quality from all nerve segments could be regarded with the same level of confidence. Figure 2 displays the proportions of median, radial and ulnar nerve segments for the Tensor and iFOD2 algorithms where acquisition was ‘full’ (where the algorithm successfully seeded 5000 streamlines), ‘partial’ (where the algorithm generated between 1 and 4999 streamlines before the maximum number of sampling attempts was reached), ‘absent’ (where the algorithm generated no streamlines) or where the algorithm reported an error. Median and ulnar nerve tractography was at least partially successful in over 80% of imaged nerve segments, whereas radial nerve tractography was significantly less successful in comparison (*p* < 0.0001), with only 37.5% and 62.5% of nerve segments yielding at least partial data using the Tensor and iFOD2 algorithms respectively.

Additionally, tractography of the shorter (distal, middle and proximal third) nerve segments was notably more successful than when the algorithm was instructed to track the full nerve (Figure S1), where between a third and half of the seeding attempts were unsuccessful in generating data. The iFOD2 algorithm was consistently more successful than the Tensor algorithm.

As a further data quality control measure, we investigated whether MMNCB nerves with CB were harder to track than HC, as this might be expected to affect data analysis. Tractography was not significantly different in nerve segments with CB (*p* = 0.81 for Tensor and 0.59 for iFOD2 algorithms). Equally, nerves where CB was identified somewhere along the course of the nerve (for instance, a proximal ulnar segment where CB was found in the distal ulnar segment) were not harder to track than nerves without CB along the course of the nerve (Tensor *p* = 0.078, iFOD2 *p* = 0.034 with non‐affected nerves trending towards poorer acquisition). MMNCB patients' nerves in general may have been marginally harder to acquire than HC nerves (*p* = 0.013 for at least partial data acquisition).

### 3.2 Diffusion Parameters Do Not Show Consistent Changes in Areas of Proven Conduction Block on Visual Inspection

**FIGURE 3:**
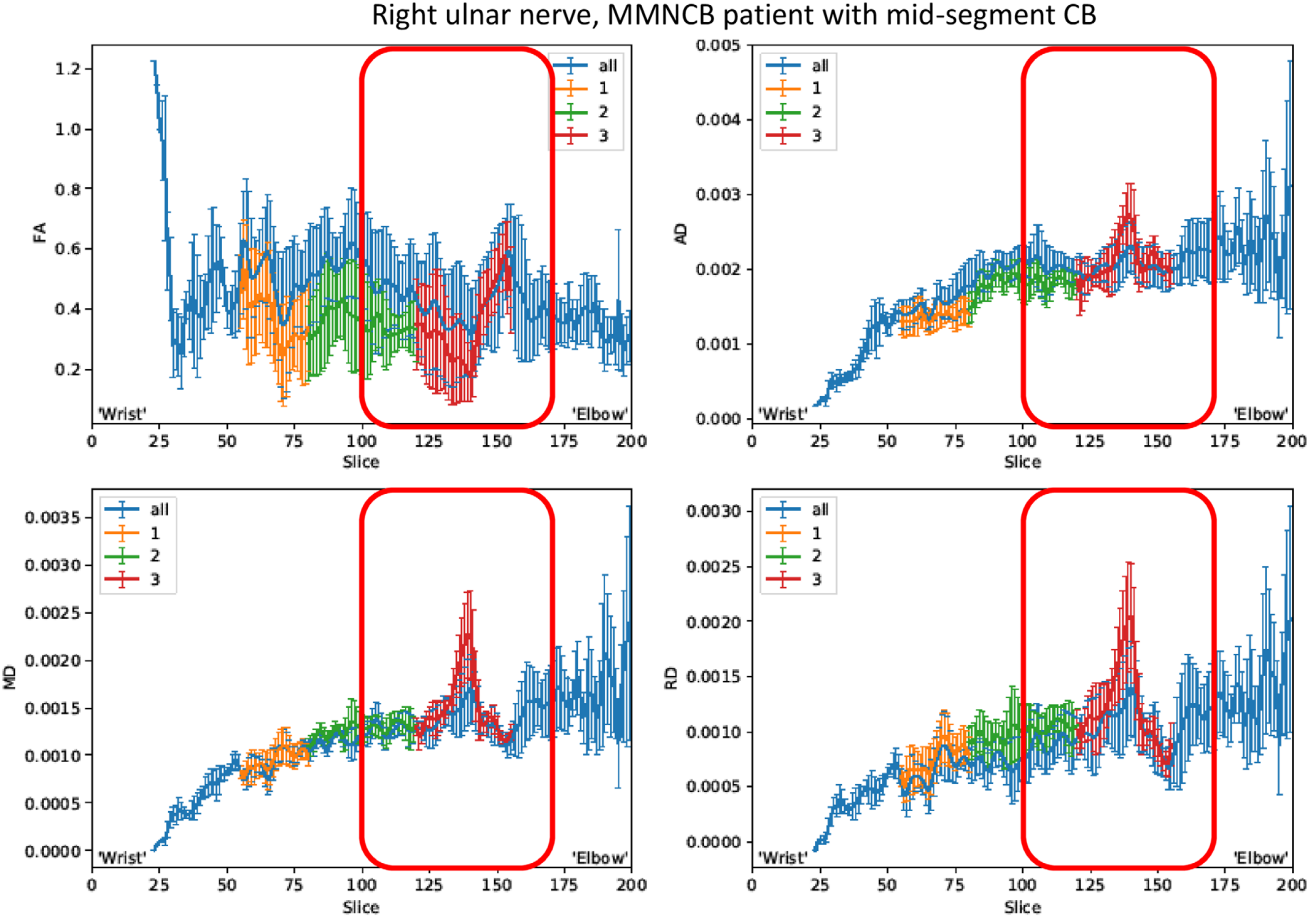
Longitudinal diffusion parameter profile of an MMNCB patient's right ulnar nerve using Tensor algorithm. Conduction block was found on nerve conduction studies between the mid‐forearm and upper third of forearm (red brackets). The horizontal trendline represents mean value along the forearm and the vertical bars represent standard deviation. A possible slight spike is seen in the shorter proximal segment on AD, MD and RD with a possible local reduction in FA (in red), but the changes are poorly reflected in the full nerve segment (blue). AD, axial diffusivity; FA, fractional anisotropy; MD, mean diffusivity; RD, radial diffusivity.

**FIGURE 4:**
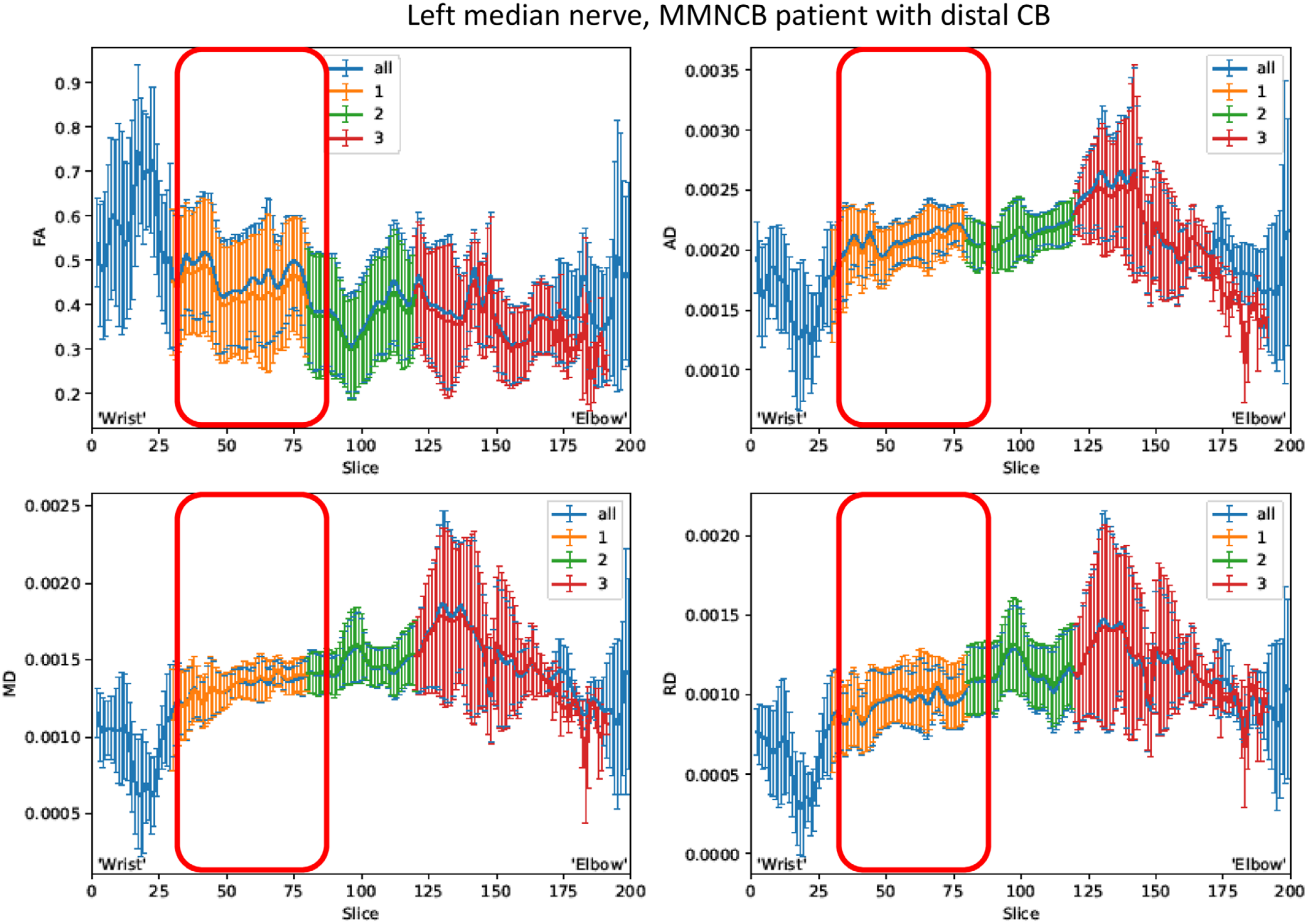
Longitudinal diffusion parameter profile of an MMNCB patient's left median nerve using the iFOD2 algorithm. Conduction block was found on nerve conduction studies, starting 30 mm from the wrist crease and stretching over 55 mm (red brackets). The horizontal trendline represents mean value along the forearm and the vertical bars represent standard deviation. No evident diffusion parameter changes were seen in the area of interest. AD, axial diffusivity; FA, fractional anisotropy; MD, mean diffusivity; RD, radial diffusivity.

**FIGURE 5:**
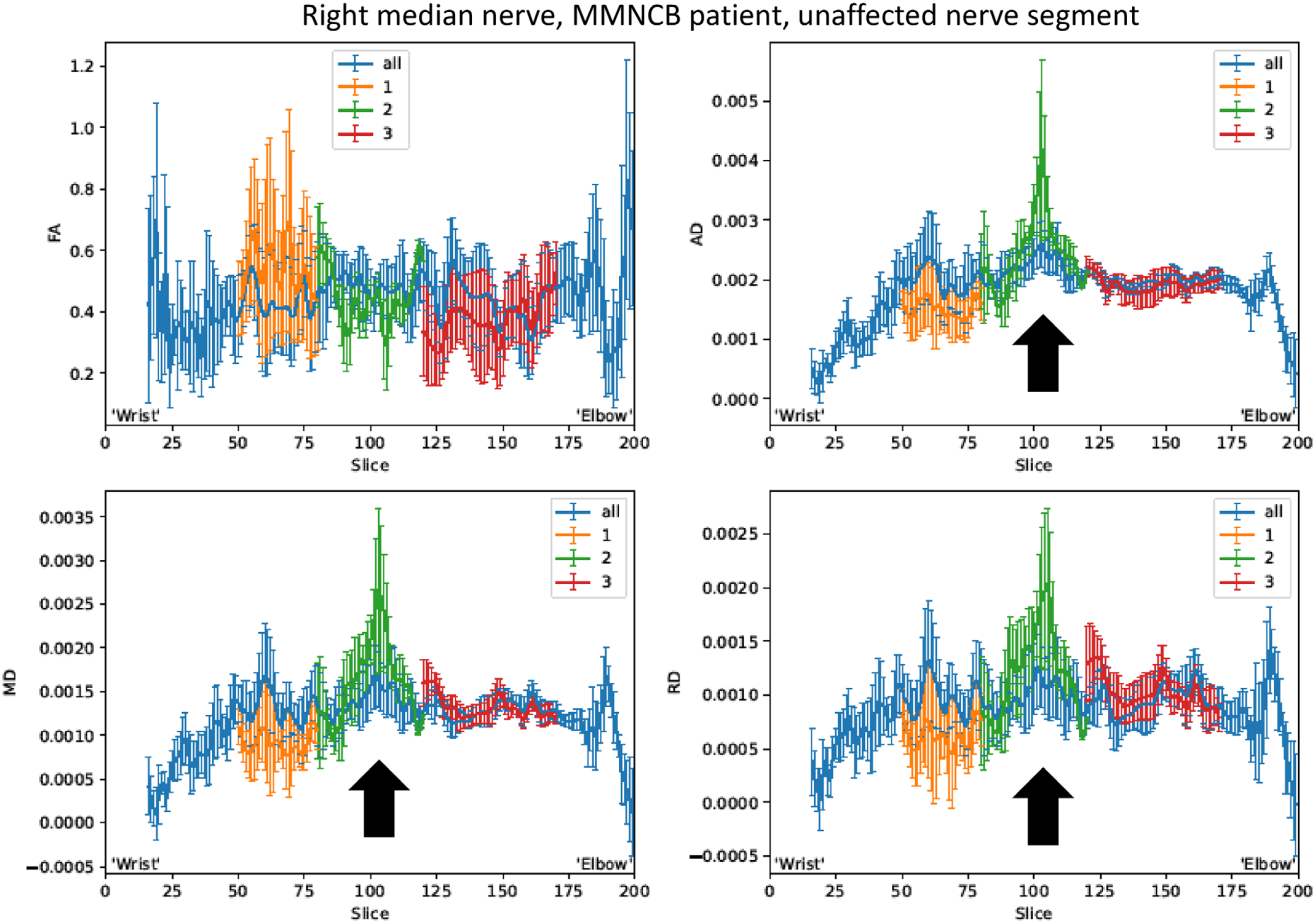
Longitudinal diffusion parameter profile of an MMNCB patient's unaffected right median using Tensor algorithm. The horizontal trendline represents mean value along the forearm and the vertical bars represent standard deviation. Despite this nerve displaying no conduction block on nerve conduction studies, a spike in AD, MD and RD (arrows) can be seen in the middle segment (green). AD, axial diffusivity; FA, fractional anisotropy; MD, mean diffusivity; RD, radial diffusivity.

**FIGURE 6:**
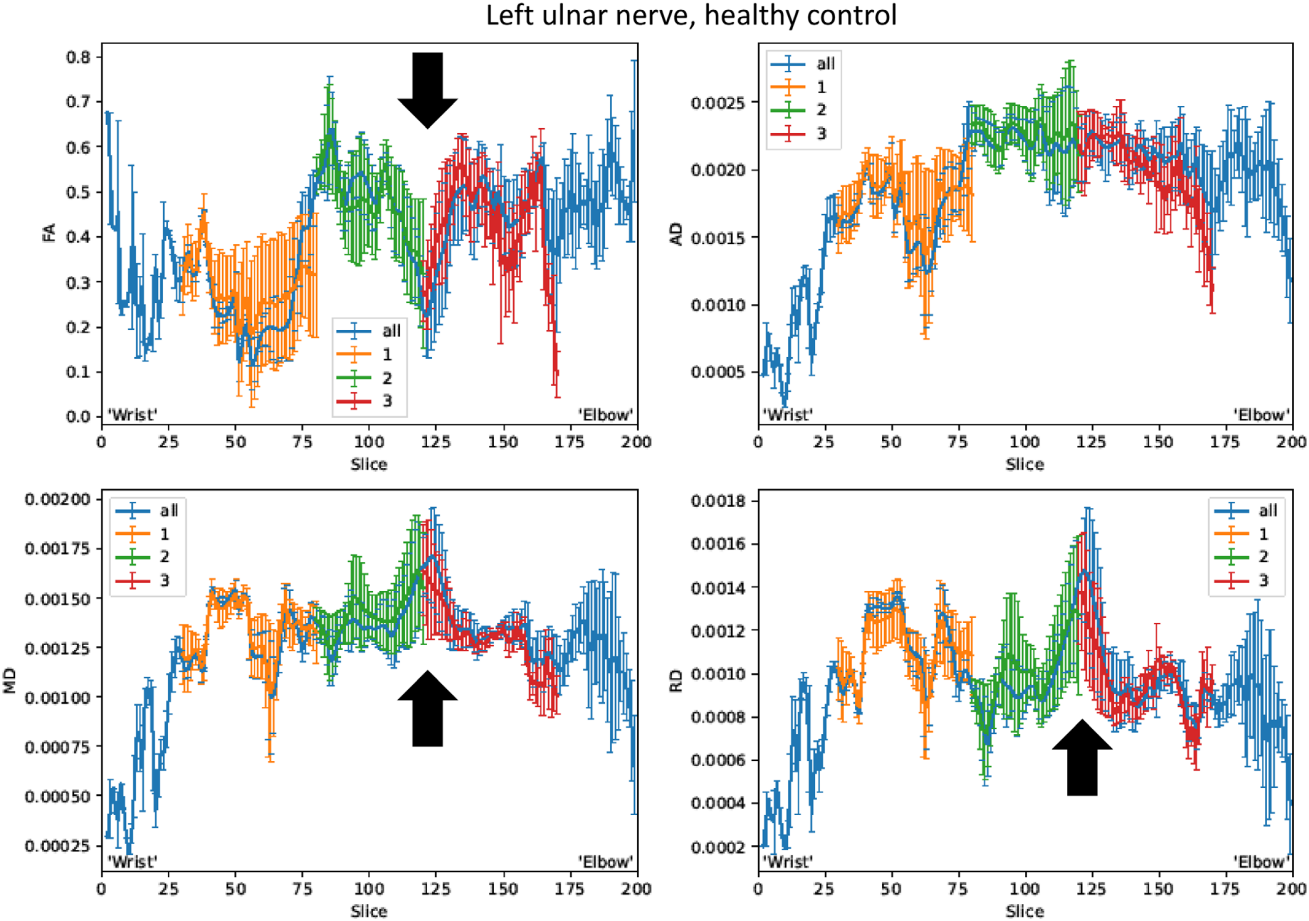
Longitudinal diffusion parameter profile of a healthy control left ulnar nerve using Tensor algorithm. The horizontal trendline represents mean value along the forearm and the vertical bars represent standard deviation. Despite this being a healthy control nerve, a spike in FA, MD and RD (arrows) can be seen in the middle segment (green). AD, axial diffusivity; FA, fractional anisotropy; MD, mean diffusivity; RD, radial diffusivity.

Composite longitudinal profiles of each subject's right and left median, ulnar and radial nerves were created, displaying the FA, AD, MD and RD diffusion parameters for the three (distal, middle and proximal) nerve segments as well as the full nerve profile. These profiles were scrutinised for consistent changes within the areas where NCS identified CB (see Figures 3, 4, 5, 6).

Figures 3, 4, 5, 6 display examples of composite nerve segment profiles, including where localised changes were identified in areas of neurophysiological CB, where no identifiable changes were seen and where changes *were* seen in normal nerves. Overall, while a proportion of nerve profiles displayed some potential areas of change in the region where CB was identified on NCS (elevation in AD, MD and RD and reduction in FA) (Figure 3), there were no identifiable changes in diffusion parameters in other regions where CB was confirmed (Figure 4), and in some cases, localised alteration of diffusion parameters was seen where no CB was found on NCS (Figures 5 and 6).

The overall characteristics and accuracy of this approach are summarised in Table 2. Visual inspection of the diffusion parameter profiles was of limited value in identifying CB, with low sensitivity (28.6% for iFOD2 and 42.9% for Tensor algorithm) and positive predictive value (44.4% for iFOD2; 54.5% for Tensor algorithm). Crucially, the localised pattern of changes (elevation in AD, MD and RD and reduction in FA) seen in some areas of NCS‐confirmed CB occurred both in MMNCB patients where no block was found on NCS (Figure 4) and HCs (Figure 5), making it highly unlikely that these correlated with any pathology.

**TABLE 2:** Summary characteristics of longitudinal diffusion parameter profiles of individual nerves from the entire cohort.

Algorithms	iFOD2		Tensor	
	Number of profiles	% total	Number of profiles	% total
Total number of nerve profiles	120	—	120	—
Error/no data	9/120	7.5	16/120	13.3
Incomplete data (e.g., one or more nerve segment missing)	30/120	25	44/120	36.7
Full data available	81/120	67.5	60/120	50
*Nerves with CB identified on NCS*				
Total number	14/120	11.7	14/120	11.7
Pattern of decreased FA and elevated AD/MD/RD on visual inspection at site of block	4/120	3.3	6/120	5
No identified changes	10/120	8.3	8/120	6.7
Sensitivity	28.6%		42.9%	
*Nerves without CB (MMNCB and HC)*				
Total number	97/120	80.8	90/120	75
No identified changes	92/120	76.7	85/120	70.8
Pattern of decreased FA and elevated AD/MD/RD	5/120	4.2	5/120	4.2
Specificity	94.8%		94.4%	

Abbreviations: CB, conduction block; HC, healthy control; MMNCB, multifocal motor neuropathy with conduction block; NCS, nerve conduction studies.

### 3.3 Analysis of Pooled Diffusion Parameter Values Shows No Definite Statistically Significant Subgroup Differences and Marked Overlap Between Groups

Previous studies have reported differences in diffusion parameters by assigning each whole nerve segment a single mean FA/AD/MD/RD value, and analysing the average and standard deviation of these summary values. We have replicated this for each individual nerve segment (left and right median, ulnar and radial nerve split into whole nerve and equal distal, middle and proximal thirds) where the algorithm fully acquired 5000 tracts, and have found no statistical differences between MMN and HC nerve segments, or between nerve segments with CB within the segment and comparable HC nerve segments when corrected for multiple comparisons (Tables S2 and S3). Finding no differences, we proceeded to a more detailed analysis of pooled diffusion parameter data in a subsection of nerve segments.

The distal median and proximal radial nerve segments were selected as representative subsets of the entire cohort. Nerve segments were analysed separately because no existing data confirm that normal nerves have uniform diffusion characteristics. The pooled data were analysed for potential differences compared to known clinical characteristics, including whether nerves were from HCs or MMNCB patients, whether the nerve was from the worse affected MMNCB arm, whether the nerve itself was affected by CB, and active Ig treatment.

**FIGURE 7:**
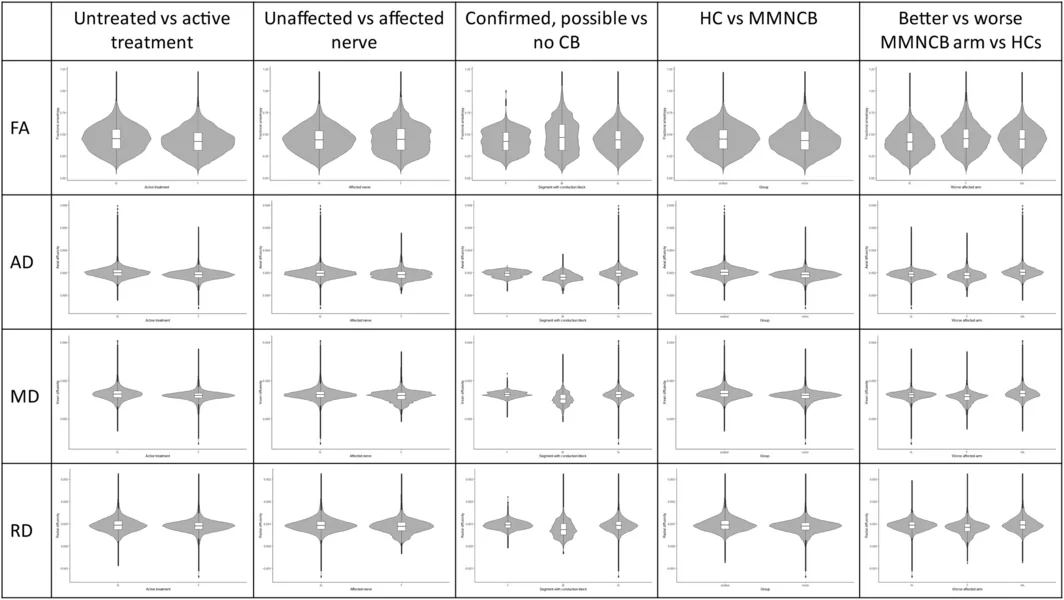
Distribution of diffusion parameters from the distal third of the median nerve from MMNCB patients and healthy controls using the iFOD2 algorithm. The plots displayed are violin plots with overlaid box plots representing the median, first and third quartile values. Note that each plot represents > 1 × 10^6^ diffusion parameter values. The graphs demonstrate the marked overlap in diffusion parameter values regardless of presence or absence of conduction block, treatment status and patient group, with long ‘tails’ of outlying values at both extremes in most subgroups. AD, axial diffusivity; CB, conduction block; FA, fractional anisotropy; HC, healthy controls; MD, mean diffusivity; MMNCB, multifocal motor neuropathy with conduction block; RD, radial diffusivity.

Table S4 details the summary characteristics of the pooled diffusion parameter data. Statistical comparisons of the mean FA, AD, MD and RD values for individual nerve segments revealed only a few *p* values of modest significance (0.001 < *p* < 0.05), and these were mainly between MMNCB and HC nerves; none retained significance on Bonferroni correction for repeated comparisons. Plotting the distribution of the diffusion parameters across cohort subgroups (Figure 7 and Figures S2–S4) demonstrates the prominent overlap regardless of clinical characteristics, making it impossible to create practical cut‐offs for normal values.

We explored the possibility that the extended upwards ‘tails’ of outlying FA, AD, MD and RD values, which appear to occur in most subgroups but seemed to be absent in nerve segments with CB and in nerves with CB along their course (Figure 7 and Figures S2–S4), might provide a potential ‘normal’ value that distinguished diseased and healthy nerves. However, it was not possible to identify a sufficiently discriminatory individual diffusion parameter cut‐off value.

### 3.4 Areas of Nerve STIR Hyperintensity Are Seen in Some Individuals With MMNCB, but Do Not Colocalise With Areas of Electrophysiological Conduction Block

Qualitative evaluation of STIR imaging revealed that four of the 20 image sets had either some movement artefact or other technical issues that were judged to impede the analysis in at least some parts of the median or ulnar nerve. Only one of these was in a region where the area of CB was within the region of poor imaging quality.

Forearm muscle STIR denervation changes were identified in three subjects, all of whom had MMNCB. All three had denervation changes in the arm where CB was identified, although one of these subjects had bilateral arm muscle denervation changes whereas CB was only identified on one side.

All 40 nerves (bilateral median and ulnar) from the 10 control subjects were visualised adequately. Nine subjects were judged to have normal STIR nerve signal. Only two nerves were felt to have some STIR hyperintensity: a focal segment in the proximal ulnar and an equivocal area of hyperintensity in the middle segment of the median nerve, both from the same control subject. As controls did not undergo NCS, it is unclear whether the proximal ulnar signal change may have been subclinical ulnar nerve compression at the cubital tunnel. Amongst the 10 MMNCB subjects, six were felt to have some areas of nerve hyperintensity. One subject's left ulnar nerve was not adequately visualised through its entire length due to artefact. Fourteen nerves were judged to have STIR hyperintensity and of these seven were in nerves with CB identified on NCS. However, only three of these nerves had an area of STIR hyperintensity which matched the neurophysiological localisation of CB. Five nerves with CB had no STIR hyperintensity identified on consensus review. Of the 14 MMNCB nerves deemed to have STIR hyperintensity, nine had focal hyperintensity (five proximal and four midsegment), four had a multifocal pattern and one had diffuse abnormality.

Areas of STIR hyperintensity also correlated poorly with the areas on DTI longitudinal profiles (elevation in AD, MD and RD and reduction in FA), which occasionally coincided with CB.

Table 3 summarises the evaluation of STIR imaging in the patient cohort.

**TABLE 3:** Summary of STIR imaging review in nerves with and without conduction block.

		STIR normal	STIR hyperintensity	Sensitivity/specificity
MMNCB only (*n* = 39)	CB on NCS	5	7 (3 in right location)	Sensitivity 58% (25%)^a^ Specificity 74%
	No CB	20	7	
MMNCB and HC (*n* = 79)	CB on NCS	5	7 (3 in right location)	Sensitivity 58% (25%)^a^ Specificity 87%
	No CB	58	9	

Abbreviations: CB, conduction block; HC, healthy controls; MMNCB, multifocal motor neuropathy with conduction block; STIR, short *tau* inversion recovery.

^a^ The number in brackets indicates the sensitivity calculated using only the nerves where STIR hyperintensity localised to the same area of the nerve where CB was identified.

As STIR hyperintensity in MMNCB patients correlated poorly with areas of CB, we compared the distal compound muscle action potential (CMAP) amplitude reported on NCS in nerves with and without STIR abnormalities to investigate whether this might instead be a marker for axonal degeneration. There was no significant difference in CMAP amplitude between STIR‐normal and STIR‐abnormal nerves (one‐tailed *t*‐test *p* > 0.05). This, in conjunction with the observation that all but one of the STIR abnormalities were in the proximal or middle part of the nerve rather than distally, indicates that STIR abnormality is very unlikely to indicate distal axonal degeneration in this cohort.

## 4 Discussion

We have demonstrated that existing high resolution 3‐T MRI DTI techniques are not able to identify any useful characteristics of clinically relevant pathology in forearm nerves of individual patients with MMNCB at the site of CB or elsewhere; those patients were from a cohort of MMNCB patients with detailed electrophysiological localisation of block. Both DTI and STIR MRI sequences failed to capture morphologic abnormalities that could correspond with electrodiagnostic proof of motor CB in the same segment, and visual inspection of nerve morphology in the forearm segments does not appear to be accurate for either diagnosis or identification of functionally abnormal nerve segments in MMNCB patients.

The study design allowed for a high level of confidence in the localisation of CB, as most individuals had NCS on the same day as their MRI appointments. Although two patients had a short delay between NCS and imaging, both were noted to have localised changes (decreased FA and elevated AD/MD/RD) within the segment of NCS‐identified CB on at least one of the DTI algorithms, without any ‘false positive’ changes in nerves without CB, indicating that the interval delay was not likely to have affected the conclusions of the study. Whilst localised changes in individuals with MMNCB could be argued to demonstrate pathology too subtle for localisation using electrophysiological methods, the fact that these were noted in some HC nerves not at typical sites of nerve compression makes this highly unlikely.

The approach used in this study was successful at acquiring DTI data from the forearm nerves, and more successful in the median and ulnar nerves than the radial. Nerve differences could be because of the more circuitous path of the radial nerve in the forearm. The algorithm was also less successful in producing data on the full length of the forearm nerves than the shorter segments, and visual inspection of longitudinal diffusion parameter profiles also demonstrated more consistent changes in the shorter segments.

Consistently localising CB with NCS to a very short nerve segment is technically challenging. In some respects, it is surprising that surface electrophysiology can detect localised CB at all in axons affected in a patchy and heterogeneous manner. On the other hand, if there is a larger localised epineurial or perineurial ‘defect’ enabling a local block, one might be able to visualise with MRI techniques in the nerve trunk. Although inching techniques are well‐described to accurately localise ulnar nerve compression around the elbow [31], the precise localisation of CB to a segment only a centimetre or so in length was not uniformly possible within our MMNCB cohort. This led to challenges in defining exactly where a CB maximally sat, especially where it spanned adjoining segments; clinician discretion was necessary to decide on ‘possible’ CB. Areas of ‘possible’ CB as described here should not therefore be considered an intermediate between ‘definite’ CB and absence of block, rather more diffuse block. Conversely, diffusion parameters were often more obviously different between nerve segments where we defined ‘possible’ or no CB. It is possible that this reflects ‘possible’ CB being present in nerves that have a more diffuse area of nerve dysfunction, compared to a smaller, focussed electrophysiological region of dysfunction in a very short part of a single nerve segment.

Although there is considerable experience in the use of DTI in the central nervous system, the underlying pathophysiological reasons behind the patterns of observed changes in diffusion parameters continue to be disputed, as myelin disruption, oedema, gliosis and inflammation can all affect MRI diffusion characteristics [32]. In addition, there are conflicting reports on whether these parameters are increased or decreased in any given pathology [32]. Broadly, most sources suggest that FA reduces in areas of pathology [32, 33], that myelin damage increases RD, and that impaired axonal function reduces AD acutely and increases it in the chronic state [33]. These parameters are insufficiently studied in the PNS to allow for clear recognition of expected patterns with disease.

A previous study of the median and ulnar nerves in MMNCB, motor neuron disease and controls suggested that AD was preferentially reduced in MMNCB proximal nerve segments [12], but technical issues necessitating data exclusion significantly limited the amount of information obtained from individuals with disease. A further study with similar methodology indicated that pooled upper limb DTI data from the median, ulnar and radial nerves display a statistically significant reduction in FA and increase in RD in MMNCB compared to HC nerves [13]. The imaging protocol for this study captured DTI data on standardised slices in the mid‐upper arm and was designed to explore group differences in DTI parameters rather than localised changes at sites of CB in individuals of clinical utility.

The observation that the diffusion parameter distributions of nerves affected by CB appear to lack the long outlier ‘tails’ may merit further investigation in future studies. At present it is impossible to suggest a pathological basis for this observation, and using these to create individual ‘normal’ values is not possible as each ‘tail’ generally represents values from a small number of individual nerves. Whilst a composite measure utilising cut‐off values from multiple parameters might, in theory, distinguish healthy and affected nerves, we do not advocate this as a diagnostic technique as its significance remains uncertain.

Prior studies of the brachial plexus and upper arms in MMNCB [8, 11] have noted STIR hyperintensity and thickening. Studies by Haakma et al. and Foesleitner et al. also noted that T2‐measured cross‐sectional area (CSA) of the median and ulnar nerves may be increased in MMNCB compared to controls and patients with ALS [12, 13]. Whilst technical and resolution challenges meant that we were not able to comment on nerve hypertrophy in the forearm, analysis of STIR signal indicates that this modality, when interpreted by experienced neuroradiologists, is most successful at identifying *normality* in HCs. A single control subject had two nerve segments with hyperintense signal change reported, but one was in the proximal ulnar nerve where subclinical ulnar nerve compression within the cubital tunnel could be a potential explanation, and the other was equivocal rather than clearly abnormal. However, analysis of STIR hyperintensity was not sensitive at identifying MMNCB. In particular, STIR hyperintensity correlated poorly with neurophysiological areas of CB.

We considered the possibility that STIR hyperintensity was due to axonal degeneration distal to a region damaged by severe CB. Distal STIR hyperintensity is seen in animal models of sciatic nerve crush injury [34]. However, all but one of the nerves in our cohort had proximal or midsegment hyperintensity within the forearm, and distal CMAP amplitude on NCS failed to correlate with the presence or absence of STIR hyperintensity, indicating that distal axonal degeneration is unlikely to explain STIR hyperintensity in our cohort, and that STIR hyperintensity of the forearm nerves is probably not reflective of a pathological process.

The finding of muscle STIR denervation changes in some MMNCB patients suggests that this may be a useful area for further research. Neuromuscular MRI of the lower limbs to identify chronic muscle denervation changes has accumulated growing evidence as a longitudinal outcome measure in hereditary neuropathies [35, 36], and is being actively investigated in other inflammatory neuropathies. As MMNCB preferentially affects the upper limb in most cases, neuromuscular MRI of the forearms may be an interesting area for further study. The finding of forearm muscles STIR signal changes may also have affected the blinding of the neuroradiologists, limiting the objective evaluation of STIR changes in the forearm nerves.

In this study, quantitative or semi‐quantitative analysis of STIR changes was not feasible due to the inter‐subject variation in imaging quality, so only a qualitative analysis was performed. Future studies with improved STIR sequences or Dixon‐based methods may enable more accurate semi‐quantitative or quantitative analysis of nerve signal, providing further detail into this process.

Our in‐depth analysis of nerves with CB in MMNCB using modern‐day 3‐T MR diffusion tensor techniques and tracking algorithms has demonstrated that this approach is unable to consistently and accurately identify and localise CB within individual nerve segments. The presence of some potential changes in a low proportion of affected nerves indicates that further advances in DTI techniques and analytical algorithms, potentially supplemented by improving imaging resolution using 7‐T MRI, might be more successful in identifying CB in MMNCB and other inflammatory neuropathies.

In addition to this, motor CB in MMNCB is thought to be a functional consequence of diverse pathways which limit action potential propagation, including abnormalities at the nodal or axonal level, which may not correspond to broader nerve morphological changes [37, 38, 39]. Besides this, pathological studies indicate that motor fibres contribute to a much smaller proportion of mixed upper limb axons than sensory fibres (around 10%) [40], such that a disorder exclusively affecting motor fibres such as MMNCB may not lead to clear morphological changes in mixed nerves.

There are many other potential limitations to this study. This includes technical difficulties scanning the forearm segments as evidenced by movement artefacts affecting a proportion of the scans leading to difficulty with nerve tract identification and limiting resolution. These issues appeared to pose a similar challenge in the study by Haakma et al. [12] The cohort of MMNCB patients studied was in general individuals with a long history of disease, and whether MRI signal changes exist in earlier phases of the disease that are not sustained during the long‐term is unknown.

Whilst DTI is a promising MRI technique with promising clinical applications outside of neuromuscular disease, it is currently neither sensitive nor specific enough to identify CB in MMNCB at an individual level in the forearm nerves. This study exemplifies the challenges currently limiting the translation of MRI from a research tool into a diagnostic clinical test in neuropathy, and why clinicians should exercise caution in utilising MRI findings in neuropathy diagnosis. At present we are unable to provide any support for the diagnostic utility of MRI in MMNCB and suggest caution in considering its inclusion in the diagnostic criteria for MMNCB and other inflammatory neuropathies, where more research is required.

## Funding

This work was supported by Muscular Dystrophy UK, 18GRO‐PG12‐0230.

## Conflicts of Interest

The authors declare no conflicts of interest.

## Supporting information


**Table S1:** Detailed breakdown of individual nerve segments affected by CB in MMNCB patients.
**Table S2:** Averaged values of each diffusion parameter within individual nerve segments for nerve segments fully acquired by the Tensor algorithm (track count = 5000; 320 observations total).
**Table S3:** Averaged values of each diffusion parameter within individual nerve segments for nerve segments fully acquired by the iFOD2 algorithm (track count = 5000; 344 observations total).
**Table S4:** Summary of diffusion parameter values along the distal median and proximal radial nerve segments, split by clinical and electrophysiological characteristics.
**Figure S1:** Proportions within the entire cohort from the distal, middle, proximal or full nerve segments with full, partial or no (absent/error) acquisition using the Tensor (A) and iFOD2 (B) algorithms.
**Figure S2:** Distribution of diffusion parameters from the distal third of the median nerve from MMNCB patients and healthy controls using the Tensor algorithm.
**Figure S3:** Distribution of diffusion parameters from the proximal third of the radial nerve from MMNCB patients and healthy controls using the iFOD2 algorithm.
**Figure S4:** Distribution of diffusion parameters from the proximal third of the radial nerve from MMNCB patients and healthy controls using the Tensor algorithm.

## Data Availability

The data that support the findings of this study are available from the corresponding author upon reasonable request.
