# Supplementary material for: High Resolution 3‐T Magnetic Resonance Tractography Cannot Localise Conduction Block in Multifocal Motor Neuropathy With Conduction Block
Source: J Peripher Nerv Syst. 2026 Sep 13;31(3):e70164. doi: 10.1111/jns.70164 (PMC13572773; doi:10.1111/jns.70164)
Supplement: Supplementary file 1 [file JNS-31-0-s001.doc]

**SUPPLEMENTARY MATERIALS**

**Supplementary Table 1: Detailed breakdown of individual nerve segments affected by CB in MMNCB** patients

| **Patient** | **Age** | **Disease duration** | **Ongoing Ig** | **MMN-RODS post-treatment** | **Dominant arm** | **GM1 ganglioside status** | **Nerve with CB** | **Segment of forearm nerve affected** |
| --- | --- | --- | --- | --- | --- | --- | --- | --- |
| A | 68 | >15 years | Y | 43/50 | Right | Unknown | Right ulnar | Distal third |
| B | 51 | 9 years | Y | 39/50 | Right | Negative | Right ulnar | Proximal third (just below elbow) |
| C | 65 | >15 years | Y | 42/50 | Right | Positive (IgM >1:150,000) | Right ulnar | Proximal third (60mm below elbow) |
| D | 55 | 11 years | Y | 48/50 | Right | Negative | Right ulnar | Proximal half |
|  |  |  |  |  |  |  | Right median | Diffuse |
|  |  |  |  |  |  |  | Right radial | Unable to localise |
| E | 31 | 6 years | Y | 41/50 | Right | Negative | Left median | 30mm from wrist crease stretching 55mm proximally |
|  |  |  |  |  |  |  | Right median | 70mm from wrist crease stretching 75mm proximally |
|  |  |  |  |  |  |  | Right ulnar | 70mm from wrist crease stretching 80mm proximally |
| F | 55 | 18 years | Y | 43/50 | Right | Unknown | Right ulnar | Between mid-forearm and proximal third |
| G | 43 | 13 years | Y | 45/50 | Right | Unknown | Left median | Distal half |
| H | 18 | 1 year | Y | 32/50 | Right | Unknown | Right radial | Proximal third |
| I | 43 | 15 years | Y | 45/50 | Left | Unknown | Left median | Middle (and probably distal) third |
| J | 33 | 15 years | Y | 32/50 | Right | Negative | Right median | Middle third (and probably proximal third) |
|  |  |  |  |  |  |  | Right radial | Unable to localise |

*CB: conduction block; Ig: immunoglobulin; MMN-RODS: multifocal motor neuropathy Rasch-built overall disability scale; MMNCB: multifocal motor neuropathy with conduction block*

**Supplementary Table 2: Averaged** values of each diffusion parameter within individual nerve segments for nerve segments fully acquired by the Tensor algorithm (track count = 5000; 320 observations total)

| Nerve segment (number of observations) | | | Mean of diffusion parameter (SD) | | | | | | | | | | | |
| --- | --- | --- | --- | --- | --- | --- | --- | --- | --- | --- | --- | --- | --- | --- |
|  |  |  | FA_mean­_ (x10^-2^) | | | AD_mean­_ (x10^-3^ mm^2^/s) | | | MD_mean_ (x10^-3^ mm^2^/s) | | | RD_mean_ (x10^-3^ mm^2^/s) | | |
|  |  |  | MMNCB | HC | CB | MMNCB | HC | CB | MMNCB | HC | CB | MMNCB | HC | CB |
| Median (140) | Whole nerve (31) | Left (15) | 42.1 (6.0) | 44.3 (6.9) | 40.9 (1.1) | 1.95 (0.18) | 2.14 (0.12) | 1.96 (0.20) | 1.30 (0.11) | 1.39 (0.14) | 1.32 (0.15) | 0.98 (0.13) | 1.02 (0.16) | 1.00 (0.13) |
|  |  |  |  |  |  | *P=0.025* | |  |  |  |  |  |  |  |
|  |  | Right (16) | 41.7 (3.6) | 43.2 (5.0) | 43.9 (4.1) | 1.87 (0.14) | 2.02 (0.17) | 1.79 (0.22) | 1.25 (0.12) | 1.33 (0.12) | 1.17 (0.15) | 0.94 (0.11) | 0.99 (0.12) | 0.86 (0.13) |
|  | Distal (38) | Left (20) | 39.5 (7.3) | 41.1 (9.5) | 38.2 (9.7) | 1.74 (0.26) | 1.96 (0.22) | 1.81 (0.31) | 1.18 (0.12) | 1.32 (0.18) | 1.24 (0.10) | 0.90 (0.08) | 0.99 (0.19) | 0.95 (0.01) |
|  |  | Right (18) | 46.2 (8.2) | 44.2 (9.9) | NA (NA) | 1.77 (0.32) | 2.10 (0.32) | NA (NA) | 1.14 (0.23) | 1.37 (0.21) | NA (NA) | 0.84 (0.20) | 1.00 (0.21) | NA (NA) |
|  | Middle (35) | Left (18) | 41.0 (5.4) | 40.6 (6.6) | 32.5 (NA) | 1.99 (0.21) | 2.18 (0.20) | 1.92 (NA) | 1.34 (0.15) | 1.48 (0.19) | 1.42 (NA) | 1.02 (0.14) | 1.12 (0.20) | 1.17 (NA) |
|  |  | Right (17) | 42.9 (4.1) | 42.0 (4.9) | 44.7 (10.0) | 2.14 (0.20) | 2.14 (0.16) | 2.25 (0.16) | 1.41 (0.13) | 1.42 (0.12) | 1.45 (0.03) | 1.05 (0.11) | 1.06 (0.12) | 1.06 (0.12) |
|  | Proximal (37) | Left (19) | 40.0 (10.1) | 41.3 (6.8) | NA (NA) | 2.08 (0.37) | 2.21 (0.21) | NA (NA) | 1.41 (0.23) | 1.47 (0.11) | NA (NA) | 1.08 (0.20) | 1.11 (0.10) | NA (NA) |
|  |  | Right (18) | 39.2 (11.4) | 38.9 (3.4) | NA (NA) | 1.95 (0.17) | 2.00 (0.14) | NA (NA) | 1.33 (0.10) | 1.36 (0.08) | NA (NA) | 1.02 (0.16) | 1.04 (0.07) | NA (NA) |
| Ulnar (129) | Whole nerve (23) | Left (9) | 40.6 (10.5) | 41.8 (7.1) | NA (NA) | 1.98 (0.15) | 1.85 (0.12) | NA (NA) | 1.34 (0.19) | 1.23 (0.08) | NA (NA) | 1.02 (0.23) | 0.92 (0.10) | NA (NA) |
|  |  | Right (14) | 38.7 (6.5) | 40.0 (7.6) | 38.0 (7.0) | 2.01 (0.25) | 2.04 (0.45) | 1.95 (0.12) | 1.39 (0.23) | 1.38 (0.32) | 1.35 (0.18) | 1.08 (0.23) | 1.05 (0.27) | 1.05 0.20) |
|  | Distal (35) | Left (18) | 35.6 (3.6) | 33.6 (11.6) | NA (NA) | 1.91 (0.20) | 1.85 (0.57) | NA (NA) | 1.36 (0.13) | 1.33 (0.43) | NA (NA) | 1.09 (0.11) | 1.07 (0.04) | NA (NA) |
|  |  | Right (17) | 40.1 (9.0) | 40.6 (7.2) | 31.7 (NA) | 1.91 (0.52) | 2.01 (0.54) | 1.99 (NA) | 1.31 (0.41) | 1.34 (0.33) | 1.45 (NA) | 1.01 (0.37) | 1.01 (0.24) | 1.18 (NA) |
|  | Middle (35) | Left (16) | 33.5 (6.3) | 39.3 (7.8) | NA (NA) | 2.07 (0.40) | 1.89 (0.34) | NA (NA) | 1.49 (0.29) | 1.28 (0.20) | NA (NA) | 1.20 (0.25) | 0.97 (0.16) | NA (NA) |
|  |  |  |  |  |  |  |  |  | *p=0.046* | |  | *p=0.046* | |  |
|  |  | Right (19) | 37.3 (3.5) | 38.4 (7.2) | 35.3 (0.16) | 2.05 (0.43) | 1.95 (0.51) | 2.16 (0.48) | 1.42 (0.31) | 1.33 (0.33) | 1.53 (0.35) | 1.11 (0.25) | 1.02 (0.25) | 1.21 (0.29) |
|  | Proximal (36) | Left (18) | 33.6 (7.1) | 35.2 (6.1) | NA (NA) | 1.98 (0.30) | 1.78 (0.31) | NA (NA) | 1.41 (0.18) | 1.25 (0.17) | NA (NA) | 1.13 (0.16) | 0.99 (0.12) | NA (NA) |
|  |  |  |  |  |  |  |  |  | *p=0.047* | |  | *p=0.031* | |  |
|  |  | Right (18) | 35.2 (6.6) | 41.3 (11.1) | 34.8 (10.7) | 1.96 (0.19) | 2.03 (0.25) | 1.92 (0.24) | 1.39 (0.14) | 1.37 (0.22) | 1.36 (0.12) | 1.10 (0.14) | 1.03 (0.25) | 1.09 (0.14) |
| Radial (50) | Whole nerve (2) | Left (1) | NA (NA) | 48.6 (NA) | NA (NA) | NA (NA) | 1.90 (NA) | NA (NA) | NA (NA) | 1.19 (NA) | NA (NA) | NA (NA) | 0.83 (NA) | NA (NA) |
|  |  | Right (1) | NA (NA) | 46.6 (NA) | NA (NA) | NA (NA) | 1.57 (NA) | NA (NA) | NA (NA) | 1.04 (NA) | NA (NA) | NA (NA) | 0.78 (NA) | NA (NA) |
|  | Distal (10) | Left (5) | 23.4 (3.9) | 42.0 (5.8) | NA (NA) | 1.42 (0.04) | 1.45 (0.08) | NA (NA) | 1.14 (0.03) | 0.97 (0.04) | NA (NA) | 1.00 (0.06) | 0.72 (0.27) | NA (NA) |
|  |  | Right (5) | 52.0 (1.3) | 39.1 (19.8) | NA (NA) | 1.64 (0.65) | 2.11 (0.89) | NA (NA) | 1.04 (0.44) | 1.50 (0.71) | NA (NA) | 0.74 (0.34) | 1.19 (0.65) | NA (NA) |
|  | Middle (20) | Left (9) | 36.0 (2.2) | 33.1 (9.7) | NA (NA) | 1.66 (0.05) | 1.62 (0.33) | NA (NA) | 1.17 (0.01) | 1.117 (0.15) | NA (NA) | 0.92 (0.007) | 0.94 (0.01) | NA (NA) |
|  |  | Right (11) | 29.9 (7.1) | 29.8 (5.3) | NA (NA) | 1.74 (0.23) | 1.92 (0.59) | NA (NA) | 1.33 (0.24) | 1.44 (0.47) | NA (NA) | 1.13 (0.25) | 1.21 (0.42) | NA (NA) |
|  | Proximal (18) | Left (9) | 30.3 (NA) | 30.6 (7.3) | NA (NA) | 2.78 (NA) | 1.71 (0.28) | NA (NA) | 2.14 (NA) | 1.27 (0.18) | NA (NA) | 1.82 (NA) | 1.05 (0.16) | NA (NA) |
|  |  | Right (9) | 30.4 (7.4) | 29.6 (4.9) | 35.6 (NA) | 1.99 (0.46) | 2.13 (0.77) | 1.67 (NA) | 1.49 (0.44) | 1.58 (0.55) | 1.18 (NA) | 1.24 (0.43) | 1.31 (0.44) | 0.93 (NA) |

*CB: conduction block; HC: healthy control; MMNCB: multifocal motor neuropathy with conduction block; NA: no SD presented as only single value available for nerve segment, or no values within this group.*

**Supplementary Table 3**: Averaged values of each diffusion parameter within individual nerve segments for nerve segments fully acquired by the iFOD2 algorithm (track count = 5000; 344 observations total)

| Nerve segment (number of observations) | | | Mean of diffusion parameter (SD) | | | | | | | | | | | |
| --- | --- | --- | --- | --- | --- | --- | --- | --- | --- | --- | --- | --- | --- | --- |
|  |  |  | FA_mean­_ (x10^-2^) | | | AD_mean­_ (x10^-3^ mm^2^/s) | | | MD_mean_ (x10^-3^ mm^2^/s) | | | RD_mean_ (x10^-3^ mm^2^/s) | | |
|  |  |  | MMNCB | HC | CB | MMNCB | HC | CB | MMNCB | HC | CB | MMNCB | HC | CB |
| Median (143) | Whole nerve (28) | Left (13) | 45.4 (3.3) | 46.0 (5.4) | 41.9 (NA) | 2.07 (0.13) | 2.13 (0.10) | 2.07 (NA) | 1.34 (0.11) | 1.37 (0.10) | 1.39 (NA) | 0.98 (0.11) | 0.99 (0.12) | 1.05 (NA) |
|  |  | Right (15) | 42.0 (3.8) | 45.2 (3.6) | 43.7 (4.7) | 1.97 (0.18) | 2.21 (0.15) | 1.95 (0.32) | 1.32 (0.13) | 1.43 (0.10) | 1.30 (0.22) | 1.00 (0.11) | 1.04 (0.10) | 0.97 (0.19) |
|  |  |  |  |  |  | *p=0.021* | |  |  |  |  |  |  |  |
|  | Distal (39) | Left (20) | 40.7 (5.3) | 45.6 (5.1) | 41.4 (2.8) | 1.81 (0.18) | 2.01 (0.14) | 1.88 (0.22) | 1.22 (0.09) | 1.30 (0.13) | 1.25 (0.11) | 0.93 (0.08) | 0.94 (0.13) | 0.93 (0.06) |
|  |  |  |  |  |  | *p=0.019* | |  |  |  |  |  |  |  |
|  |  | Right (19) | 46.3 (9.3) | 44.4 (8.1) | NA (NA) | 1.77 (0.34) | 2.09 (0.29) | NA (NA) | 1.16 (0.25) | 1.36 (0.17) | NA (NA) | 0.85 (0.22) | 1.00 (0.16) | NA (NA) |
|  |  |  |  |  |  | *p=0.027* | |  |  |  |  |  |  |  |
|  | Middle (38) | Left (20) | 39.0 (4.1) | 41.4 (5.1) | 33.2 (NA) | 1.97 (0.21) | 2.17 (0.16) | 1.82 (NA) | 1.35 (0.15) | 1.45 (0.15) | 1.35 (NA) | 1.04 (0.14) | 1.09 (0.16) | 1.11 (NA) |
|  |  |  |  |  |  | *p=0.041* | |  |  |  |  |  |  |  |
|  |  | Right (18) | 38.2 (4.4) | 40.6 (7.4) | 39.1 (9.1) | 2.04 (0.21) | 2.15 (0.30) | 2.27 (0.25) | 1.40 (0.13) | 1.44 (0.19) | 1.55 (0.05) | 1.09 (0.11) | 1.09 (0.17) | 1.20 (0.05) |
|  | Proximal (38) | Left (20) | 38.0 (7.3) | 41.6 (4.4) | NA (NA) | 2.03 (0.29) | 2.19 (0.15) | NA (NA) | 1.41 (0.16) | 1.46 (0.09) | NA (NA) | 1.10 (0.13) | 1.10 (0.08) | NA (NA) |
|  |  | Right (18) | 36.5 (7.8) | 39.7 (6.1) | NA (NA) | 2.05 (0.22) | 2.05 (0.24) | NA (NA) | 1.45 (0.17) | 1.38 (0.12) | NA (NA) | 1.15 (0.18) | 1.05 (0.08) | NA (NA) |
| Ulnar (128) | Whole nerve (19) | Left (7) | 36.9 (3.2) | 44.8 (8.6) | NA (NA) | 2.10 (0.07) | 2.05 (0.09) | NA (NA) | 1.48 (0.07) | 1.34 (0.17) | NA (NA) | 1.17 (0.08) | 0.98 (0.20) | NA (NA) |
|  |  | Right (12) | 39.0 (5.8) | 44.8 (6.6) | 39.5 (7.0) | 2.06 (0.24) | 2.24 (0.26) | 1.97 (0.17) | 1.42 (0.21) | 1.45 (0.21) | 1.35 (0.18) | 1.09 (0.20) | 1.06 (0.21) | 1.04 (0.20) |
|  | Distal (38) | Left (19) | 35.6 (6.3) | 38.4 (10.1) | NA (NA) | 1.88 (0.08) | 2.05 (0.32) | NA (NA) | 1.35 (0.08) | 1.43 (0.34) | NA (NA) | 1.08 (0.10) | 1.13 (0.36) | NA (NA) |
|  |  | Right (19) | 39.9 (8.4) | 41.3 (9.5) | 29.9 (NA) | 1.88 (0.33) | 2.07 (0.59) | 1.97 (NA) | 1.29 (0.29) | 1.38 (0.39) | 1.47 (NA) | 1.00 (0.28) | 1.04 (0.31) | 0.12 (NA) |
|  | Middle (35) | Left (17) | 32.3 (4.0) | 38.5 (8.1) | NA (NA) | 2.12 (0.17) | 2.17 (0.24) | NA (NA) | 1.56 (0.15) | 1.51 (0.28) | NA (NA) | 1.27 (0.16) | 1.17 (0.32) | NA (NA) |
|  |  | Right (18) | 37.6 (6.0) | 40.0 (6.3) | 37.1 (5.8) | 2.10 (0.36) | 2.14 (0.56) | 2.18 (0.34) | 1.46 (0.30) | 1.44 (0.36) | 1.52 (0.33) | 1.14 (0.27) | 1.09 (0.28) | 1.19 (0.32) |
|  | Proximal (36) | Left (18) | 33.7 (7.0) | 37.2 (8.1) | NA (NA) | 1.95 (0.22) | 2.01 (0.13) | NA (NA) | 1.40 (0.18) | 1.41 (0.16) | NA (NA) | 1.13 (0.18) | 1.10 (0.21) | NA (NA) |
|  |  | Right (18) | 37.0 (5.0) | 40.1 (6.5) | 38.5 (4.8) | 1.95 (0.14) | 2.03 (0.22) | 1.91 (0.18) | 1.36 (0.14) | 1.38 (0.18) | 1.31 (0.08) | 1.07 (0.14) | 1.05 (0.18) | 1.01 (0.02) |
| Radial (73) | Whole nerve (1) | Left (1) | NA (NA) | 49.3 (NA) | NA (NA) | NA (NA) | 1.97 (NA) | NA (NA) | NA (NA) | 1.23 (NA) | NA (NA) | NA (NA) | 0.85 (NA) | NA (NA) |
|  |  | Right (0) | NA (NA) | NA (NA) | NA (NA) | NA (NA) | NA (NA) | NA (NA) | NA (NA) | NA (NA) | NA (NA) | NA (NA) | NA (NA) | NA (NA) |
|  | Distal (24) | Left (12) | 26.7 (12.5) | 35.5 (10.6) | NA (NA) | 1.50 (0.24) | 1.66 (0.31) | NA (NA) | 1.20 (0.28) | 1.20 (0.25) | NA (NA) | 1.04 (0.30) | 0.97 (0.25) | NA (NA) |
|  |  | Right (12) | 32.3 (11.3) | 25.8 (17.3) | NA (NA) | 2.11 (0.55) | 2.17 (0.50) | NA (NA) | 1.61 (0.46) | 1.75 (0.50) | NA (NA) | 1.36 (0.43) | 1.54 (0.53) | NA (NA) |
|  | Middle (22) | Left (10) | 31.2 (4.1) | 33.8 (8.0) | NA (NA) | 1.76 (0.15) | 1.70 (0.27) | NA (NA) | 1.32 (0.17) | 1.23 (0.18) | NA (NA) | 1.10 (0.19) | 0.99 (0.18) | NA (NA) |
|  |  | Right (12) | 29.9 (3.8) | 31.5 (7.5) | NA (NA) | 2.05 (0.37) | 2.24 (0.65) | NA (NA) | 1.55 (0.33) | 1.68 (0.56) | NA (NA) | 1.30 (0.30) | 1.40 (0.53) | NA (NA) |
|  | Proximal (26) | Left (13) | 27.9 (7.9) | 33.2 (6.3) | NA (NA) | 1.99 (0.38) | 1.77 (0.26) | NA (NA) | 1.55 (0.40) | 1.29 (0.19) | NA (NA) | 1.33 (0.42) | 1.04 (0.17) | NA (NA) |
|  |  | Right (13) | 25.4 (6.4) | 29.6 (4.4) | 34.6 (NA) | 2.09 (0.62) | 2.53 (0.52) | 1.77 (NA) | 1.63 (0.34) | 1.89 (0.38) | 1.26 (NA) | 1.39 (0.38) | 1.57 (0.32) | 0.10 (NA) |

*CB: conduction block; HC: healthy control; MMNCB: multifocal motor neuropathy with conduction block; NA: no SD presented as only single value available for nerve segment, or no values within this group.*

**Supplemental Table 4: Summary of diffusion parameter values along the distal median and proximal radial nerve segments, spli**t by clinical and electrophysiological characteristics.

| **Segment** | **Algorithm** | **Diffusion parameter** | **Mean values (SD)** | | | | | | | | | | | | | | | |
| --- | --- | --- | --- | --- | --- | --- | --- | --- | --- | --- | --- | --- | --- | --- | --- | --- | --- | --- |
|  |  |  | Group | | | Segment with CB | | | | Nerve with CB along course | | | On Ig treatment | | | Worse affected arm (MMNCB only) | | |
|  |  |  | MMN | Control | P value | Definite | Possible | None | P value | Yes | No | P value | Yes | No | P value | Yes | No | P value |
| **Distal median** | **iFOD2** | AD | 0.0018 (0.0004) | 0.0021 (0.0005) | 0.0012* | 0.0019 (0.0003) | 0.0010 (0.0004) | 0.0020 (0.0005) | ns | 0.0018 (0.0005) | 0.0020 (0.0004) | ns | 0.0018 (0.0004) | 0.0020 (0.0004) | ns | 0.0017 (0.0005) | 0.0019 (0.0003) | ns |
|  |  | FA | 0.436 (0.155) | 0.456 (0.145) | ns | 0.418 (0.126) | 0.472 (0.192) | 0.440 (0.148) | ns | 0.449 (0.163) | 0.440 (0.148) | ns | 0.422 (0.148) | 0.451 (0.150) | ns | 0.461 (0.164) | 0.415 (0.143) | ns |
|  |  | MD | 0.0012 (0.0003) | 0.0013 (0.0003) | ns | 0.0013 (0.0002) | 0.0010 (0.0003) | 0.0013 (0.0003) | ns | 0.0012 (0.0004) | 0.0013 (0.0003) | ns | 0.0012 (0.0003) | 0.0013 (0.0003) | ns | 0.0011 (0.0004) | 0.0012 (0.00002) | ns |
|  |  | RD | 0.00009 (0.0003) | 0.00010 (0.0003) | ns | 0.0009 (0.0002) | 0.0008 (0.0004) | 0.0009 (0.0003) | ns | 0.0009 (0.0004) | 0.0009 (0.0003) | ns | 0.0009 (0.0003) | 0.0009 (0.0003) | ns | 0.0009 (0.0002) | 0.0009 (0.0003) | ns |
|  | **Tensor** | AD | 0.0018 (0.0004) | 0.0020 (0.0004) | 0.017* | 0.0019 (0.0003) | 0.0016 (0.0004) | 0.0020 (0.0004) | ns | 0.0018 (0.0004) | 0.0019 (0.0004) | ns | 0.0018 (0.0004) | 0.0020 (0.0004) | ns | 0.0017 (0.0005) | 0.0018 (0.0003) | ns |
|  |  | FA | 0.435 (0.156) | 0.429 (0.14) | ns | 0.398 (0.138) | 0.491 (0.208) | 0.430 (0.144) | ns | 0.452 (0.178) | 0.428 (0.144) | ns | 0.412 (0.145) | 0.443 (0.151) | ns | 0.455 (0.173) | 0.419 (0.139) | *ns* |
|  |  | MD | 0.0012 (0.0003) | 0.0014 (0.0003) | ns | 0.0013 (0.0001) | 0.00098 (0.0003) | 0.0013 (0.0003) | ns | 0.0011 (0.0003) | 0.0013 (0.0003) | ns | 0.0012 (0.0003) | 0.0013 (0.0003) | ns | 0.0011 (0.0003) | 0.0012 (0.0002) | ns |
|  |  | RD | 0.0009 (0.0003) | 0.0010 (0.0003) | 0.033* | 0.0010 (0.0002) | 0.0007 (0.0003) | 0.0010 (0.0003) | ns | 0.0008 (0.0003) | 0.0010 (0.0003) | ns | 0.0009 (0.002) | 0.0010 (0.0003) | ns | 0.0008 (0.0003) | 0.0009 (0.0002) | ns |
| **Proximal radial** | **iFOD2** | AD | 0.0020 (0.0008) | 0.0021 (0.0008) | ns | 0.0018 (0.0003) | 0.0018 (0.0005) | 0.0021 (0.0008) | ns | 0.0018 (0.0004) | 0.0021 (0.0008) | ns | 0.0022 (0.0009) | 0.0020 (0.0008) | 0.027* | 0.0020 (0.0008 | 0.0020 (0.0008) | ns |
|  |  | FA | 0.271 (0.112) | 0.322 (0.12) | 0.0042* | 0.344 (0.111) | 0.255 (0.063) | 0.302 (0.124) | ns | 0.304 (0.103) | 0.302 (0.124) | ns | 0.273 (0.116) | 0.313 (0.124) | ns | 0.271 (0.119) | 0.292 (0.115) | ns |
|  |  | MD | 0.0016 (0.0006) | 0.0015 (0.0006) | 0.025* | 0.0013 (0.0002) | 0.0014 (0.0004) | 0.0016 (0.0006) | ns | 0.0013 (0.0003) | 0.0016 (0.0006) | ns | 0.0017 (0.0006) | 0.0015 (0.0006) | ns | 0.0016 (0.0006) | 0.0016 (0.0006) | ns |
|  |  | RD | 0.0013 (0.0005) | 0.0013 (0.0005) | ns | 0.0010 (0.0002) | 0.0012 (0.0004) | 0.0013 (0.0005) | ns | 0.0011 (0.0011) | 0.0013 (0.0005) | ns | 0.0014 (0.0006) | 0.0012 (0.0005) | ns | 0.0013 (0.0005) | 0.0014 (0.0006) | ns |
|  | **Tensor** | AD | 0.0022 (0.0007) | 0.0019 (0.0008) | ns | 0.0017 (0.0003) | 0.0020 (0.0004) | 0.0020 (0.0008) | ns | 0.0017 (0.0003) | 0.0020 (0.0008) | ns | 0.0022 (0.0007) | 0.0019 (0.0008) | ns | 0.0022 (0.0007) | 0.0017 (0.0004) | ns |
|  |  | FA | 0.306 (0.0098) | 0.305 (0.112) | *ns* | 0.355 (0.107) | 0.246 (0.069) | 0.302 (0.110) | ns | 0.353 (0.108) | 0.302 (0.110) | ns | 0.305 (0.098) | 0.305 (0.113) | *ns* | 0.304 (0.097) | 0.330 (0.110) | ns |
|  |  | MD | 0.0016 (0.0006) | 0.0014 (0.0005) | 0.0076* | 0.0012 (0.0002) | 0.0016 (0.0004) | 0.0015 (0.0006) | ns | 0.0012 (0.0002) | 0.0015 (0.0006) | ns | 0.0016 (0.0006) | 0.0014 (0.0005) | ns | 0.0016 (0.0006) | 0.0013 (0.0003) | ns |
|  |  | RD | 0.0014 (0.0005) | 0.0012 (0.0004) | ns | 0.0009 (0.0002) | 0.0014 (0.0003) | 0.0012 (0.0005) | ns | 0.0009 (0.0002) | 0.0012 (0.0005) | ns | 0.0014 (0.0005) | 0.0012 (0.0004) | ns | 0.0014 (0.0005) | 0.0010 (0.0003) | ns |

**Tests of significance comparing mean diffusion parameter values for each individual nerve segment p < 0.05. ns: non-significant (p≥0.05). AD: axial diffusivity, CB: conduction block, FA: fractional anisotropy, MD: mean diffusivity, RD: radial diffusivity, SD: standard deviation.*

**Supplemental figure 1: Proportions within the entire cohort from the distal, middle, proximal or full nerve segments with full, partial, or no (absent/error) acquisition using the Tensor (A) and iFOD2 (B) algorithms.**

Full acquisition was achieved where the algorithm successfully seeded 5,000 streamlines. Partial acquisition was defined as the algorithm seeding anywhere between 1 and 4,999 streamlines after reaching the maximum number of sampling attempts. Absent acquisition and errors resulted in no diffusion parameter information for the given nerve segment.

**Supplemental Figure 2: Distribution of diffusion parameters from the distal third of the median nerve from MMNCB patients and healthy controls using the Tensor algorithm.**

The plots displayed are violin plots with overlaid box plots representing the median, first and third quartile values. Note that each plot represents >1 x 10^6^ diffusion parameter values. The graphs demonstrate the marked overlap in diffusion parameter values regardless of presence or absence of conduction block, treatment status and patient group, with long ‘tails’ of outlying values at both extremes in most subgroups. AD: axial diffusivity; CB: conduction block; FA: fractional anisotropy; HC: healthy controls; MD: mean diffusivity; MMNCB: multifocal motor neuropathy with conduction block; RD: radial diffusivity.

**Supplemental Figure 3: Distribution of diffusion parameters from the proximal third of the radial nerve from MMNCB patients and healthy controls using the iFOD2 algorithm.**

The plots displayed are violin plots with overlaid box plots representing the median, first and third quartile values. Note that each plot represents >1 x 10^6^ diffusion parameter values. The graphs demonstrate the marked overlap in diffusion parameter values regardless of presence or absence of conduction block, treatment status and patient group, with long ‘tails’ of outlying values at both extremes in most subgroups. AD: axial diffusivity; CB: conduction block; FA: fractional anisotropy; HC: healthy controls; MD: mean diffusivity; MMNCB: multifocal motor neuropathy with conduction block; RD: radial diffusivity.

**Supplemental Figure 4: Distribution of diffusion parameters from the proximal third of the radial nerve from MMNCB patients and healthy controls using the Tensor algorithm.**

The plots displayed are violin plots with overlaid box plots representing the median, first and third quartile values. Note that each plot represents >1 x 10^6^ diffusion parameter values. The graphs demonstrate the marked overlap in diffusion parameter values regardless of presence or absence of conduction block, treatment status and patient group, with long ‘tails’ of outlying values at both extremes in most subgroups. AD: axial diffusivity; CB: conduction block; FA: fractional anisotropy; HC: healthy controls; MD: mean diffusivity; MMNCB: multifocal motor neuropathy with conduction block; RD: radial diffusivity.
